# The neuronal ceroid lipofuscinosis *Cln8 *gene expression is developmentally regulated in mouse brain and up-regulated in the hippocampal kindling model of epilepsy

**DOI:** 10.1186/1471-2202-6-27

**Published:** 2005-04-13

**Authors:** Liina Lonka, Antti Aalto, Outi Kopra, Mervi Kuronen, Zaal Kokaia, Mart Saarma, Anna-Elina Lehesjoki

**Affiliations:** 1 Neuroscience Center, University of Helsinki, Finland; 2 Folkhälsan Institute of Genetics and Department of Medical Genetics, University of Helsinki, Finland; 3 Institute of Biotechnology, University of Helsinki, Finland; 4 National Public Health Institute, Finland; 5 Laboratory of Neural Stem Cell Biology, Section of Restorative Neurology, Lund Strategic Research Center for Stem Cell Biology and Cell Therapy, University Hospital, Lund, Sweden

## Abstract

**Background:**

The neuronal ceroid lipofuscinoses (NCLs) are a group of inherited neurodegenerative disorders characterized by accumulation of autofluorescent material in many tissues, especially in neurons. Mutations in the *CLN8 *gene, encoding an endoplasmic reticulum (ER) transmembrane protein of unknown function, underlie NCL phenotypes in humans and mice. The human phenotype is characterized by epilepsy, progressive psychomotor deterioration and visual loss, while motor neuron degeneration (*mnd*) mice with a *Cln8 *mutation show progressive motor neuron dysfunction and retinal degeneration.

**Results:**

We investigated spatial and temporal expression of *Cln8 *messenger ribonucleic acid (mRNA) using *in situ *hybridization, reverse transcriptase polymerase chain reaction (RT-PCR) and northern blotting. *Cln8 *is ubiquitously expressed at low levels in embryonic and adult tissues. In prenatal embryos *Cln8 *is most prominently expressed in the developing gastrointestinal tract, dorsal root ganglia (DRG) and brain. In postnatal brain the highest expression is in the cortex and hippocampus. Expression of *Cln8 *mRNA in the central nervous system (CNS) was also analyzed in the hippocampal electrical kindling model of epilepsy, in which *Cln8 *expression was rapidly up-regulated in hippocampal pyramidal and granular neurons.

**Conclusion:**

Expression of *Cln8 *in the developing and mature brain suggests roles for Cln8 in maturation, differentiation and supporting the survival of different neuronal populations. The relevance of *Cln8 *up-regulation in hippocampal neurons of kindled mice should be further explored.

## Background

The neuronal ceroid lipofuscinoses (NCLs) comprise a group of human neurodegenerative disorders (CLN1-CLN8) characterized by epilepsy, visual failure, psychomotor deterioration and accumulation of autofluorescent lipopigment in many tissues, especially in neurons [1]. Six genes underlying human NCLs have been identified and the proteins initially characterized (reviewed in [2]). Naturally occurring mouse models exist for CLN6 and CLN8 [3-5], while mouse models for CLN1, CLN2 and CLN3 have been generated by gene targeting [6-10].

The ubiquitously expressed *CLN8 *gene encodes a transmembrane protein which localizes to the ER and the ER-Golgi intermediate compartment in non-neuronal cells and to the ER in neuronal cells [4,11,12]. Mutations in *CLN8 *result in two distinct NCL phenotypes in humans: Northern epilepsy (Progressive epilepsy with mental retardation, EPMR, OMIM 600143) described in Finnish patients, and variant late infantile onset NCL in a subset of Turkish patients [4,13]. EPMR is characterized by frequent drug-resistant epileptic seizures with onset at 5–10 years of age, followed by progressive mental retardation [14], while the Turkish patients show earlier onset and a more rapid progression [15]. In EPMR, intracellular storage material, including subunit c of the mitochonrdial ATP synthase as the main protein component, is most prominent in the CNS, especially in the third layer of the isocortex and hippocampal regions CA2-4 [16]. In the cerebral isocortex the pyramidal cells of the deeper parts of lamina III are severely ballooned, and hippocampal region CA2 shows neuronal loss and neuronophagy [16]. A frameshift mutation in mouse *Cln8 *that predicts a truncated protein underlies the phenotype of the *mnd *mouse, a naturally occuring NCL mouse model [4]. Contrary to human patients, epilepsy is not a prominent feature in *mnd*, which is characterized by progressive motor neuron dysfunction and retinal degeneration [5,17,18]. The brain appears to remain relatively intact [5,17,19]. Accumulation of subunit c of the mitochondrial ATP synthase, neurofilament redistribution in spinal motor neurons and accumulation of ubiquitin deposits are characteristic for *mnd *[20-22].

Here we characterized the spatial and temporal expression of *Cln8 *mRNA in mice. Moreover, as epilepsy is the dominant phenotype in human patients, we investigated the CNS expression of *Cln8 *mRNA in the hippocampal electrical kindling model of epilepsy in which repeated electrical stimulations trigger a progressive intensification of epileptiform responses, and kindled mice retain abnormal excitability thereafter [23,24].

## Results

### The *Cln8 *gene is ubiquitously expressed in mouse tissues

**Figure 1:**
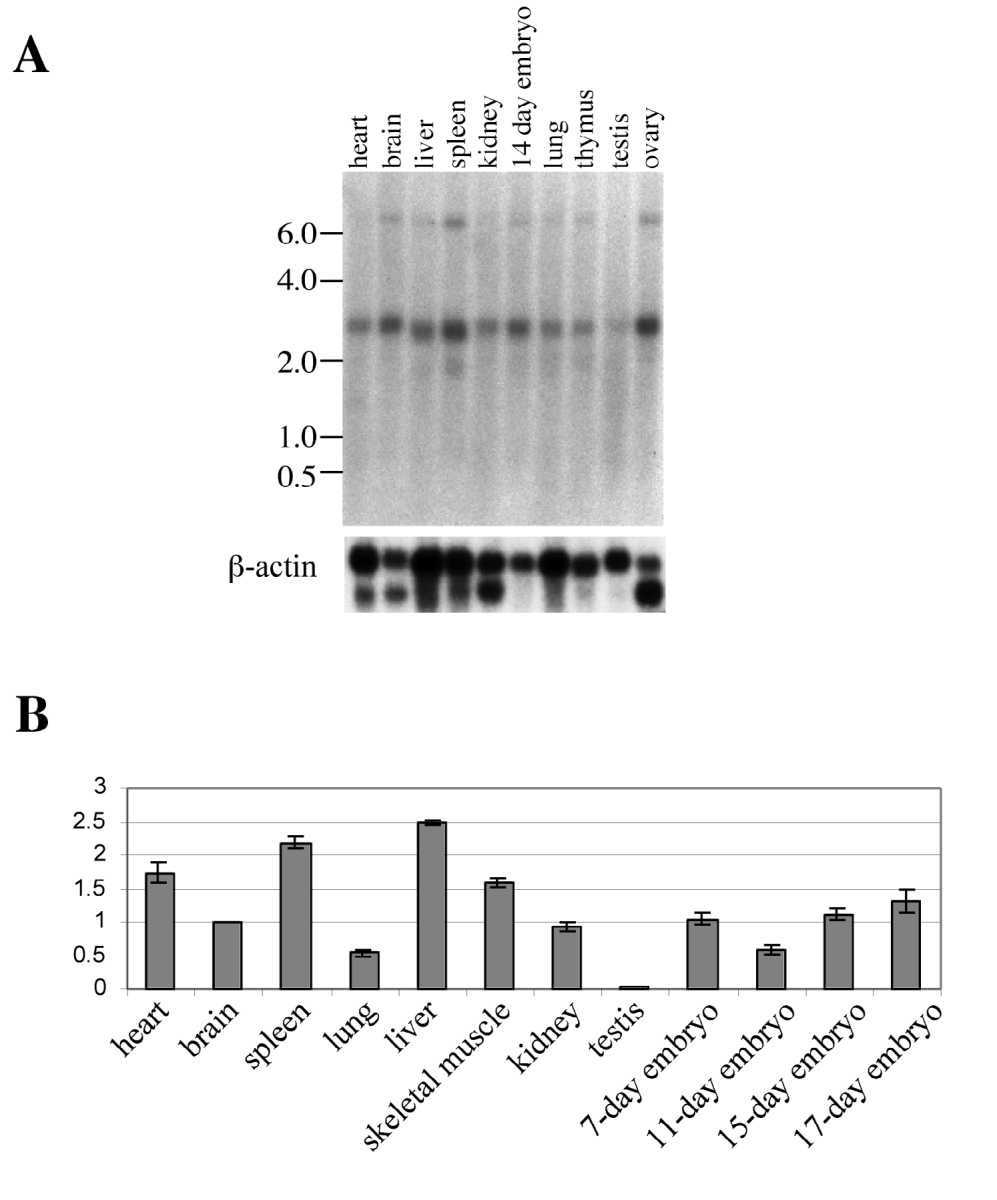
**Northern blot and RT-PCR analysis of *Cln8 *mRNA expression in mouse tissues A**: Northern blot analysis of *Cln8 *transcripts in mouse tissues. A β-actin probe was used as a control. Molecular weight marker in kb is shown on the left. **B**: Real-time quantitative RT-PCR analysis of *Cln8 *in mouse tissues. The expression of *Cln8 *in brain, given a value of 1, was used as a control. The expression of *Cln8 *in the other tissues is shown as a fold of the control.

The expression of the *Cln8 *gene in mouse tissues was first analyzed by northern blot and real-time quantitative RT-PCR analyses. In northern blot analysis one ~3 kilobase (kb) *Cln8 *specific transcript was detected in all tissues analyzed including a 14-day embryo (E14) (Fig. 1A). In addition, one ~7 kb transcript was seen in all tissues except testis and heart (Fig. 1A). In spleen, an additional transcript of ~2 kb was detected (Fig. 1A). The ~7 kb and ~2 kb transcripts were notably weaker than the ~3 kb transcript. RT-PCR analysis covering the 867 bp open reading frame of *Cln8 *resulted in a single fragment of the same size in 12 different mouse tissues (data not shown), suggesting that the different transcripts detected in the northern analysis are not due to alternative splicing in the coding region.

We then estimated the level of *Cln8 *expression using real-time quantitative RT-PCR analysis. *Cln8 *showed highest expression in liver, spleen, heart and skeletal muscle, 2.5, 2.2, 1.7 and 1.6 fold higher (respectively) than in the brain (Fig. 1B). Expression was lowest in 11-day embryo and adult kidney, lung and testis, 0.6, 0.9, 0.5 and 0.03 fold lower than in the brain (Fig. 1B). This indicates that *Cln8 *expression levels in adult mouse tissues as well as in whole mouse embryos are not dramatically different.

### *Cln8 *is expressed throughout mouse development

The tissue expression of *Cln8 *during development and brain maturation was characterized using radioactive mRNA *in situ *hybridization analysis. For this purpose, whole embryo sections from E13, E15.5 and E17 mice and brain sections from postnatal (P) P0, P5, P10 and adult mice were used and the expression of *Cln8 *was quantitated by a computer based MCID image analysis system.

**Figure 2:**
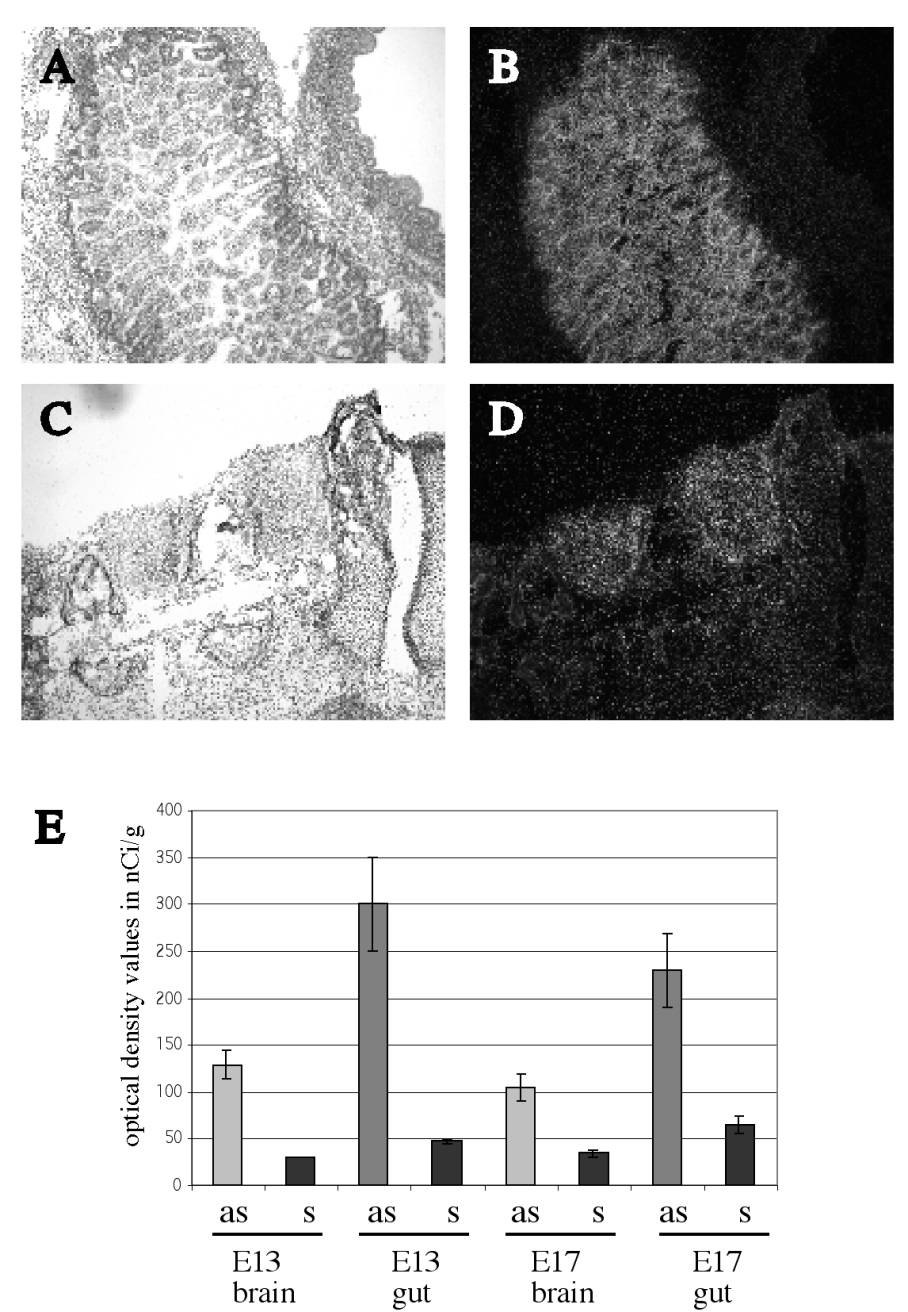
***In situ *hybridization analysis of *Cln8 *mRNA expression in mouse embryos. **In the E17 mouse embryo, developing gastrointestinal tract (A, B) and DRG (C, D), hybridized with *Cln8 *specific cRNA probe, are shown. Bright-field view of the cells is shown on the left (A, C) and dark-field detection of hybridization signals on the right (B,D). Optical density values of hybridization signals of *Cln8 *sense and antisense probes in E13 and E17 gut and brain tissues are shown in the diagram (E). Error bars represent standard error of the mean. s = sense, as = antisense

In fresh-frozen E13 and E17 embryos the overall expression of *Cln8 *was weak. In E17 the most prominent expression was detected in the developing gastrointestinal tract (Fig. 2A, 2B). In addition, there was high expression in the DRG neurons (Fig. 2C, 2D). Some *Cln8*-specific signal was also detected in the developing brain (Fig. 2E). The expression of *Cln8 *in E13 was similar to that in E17 except in the DRG neurons, where no signal was detected (data not shown). In paraffin-embedded E15.5 embryos a *Cln8*-specific signal was detected in the developing gastrointestinal tract, adrenal glands and the developing brain, especially in the cortical plate (data not shown).

**Figure 3:**
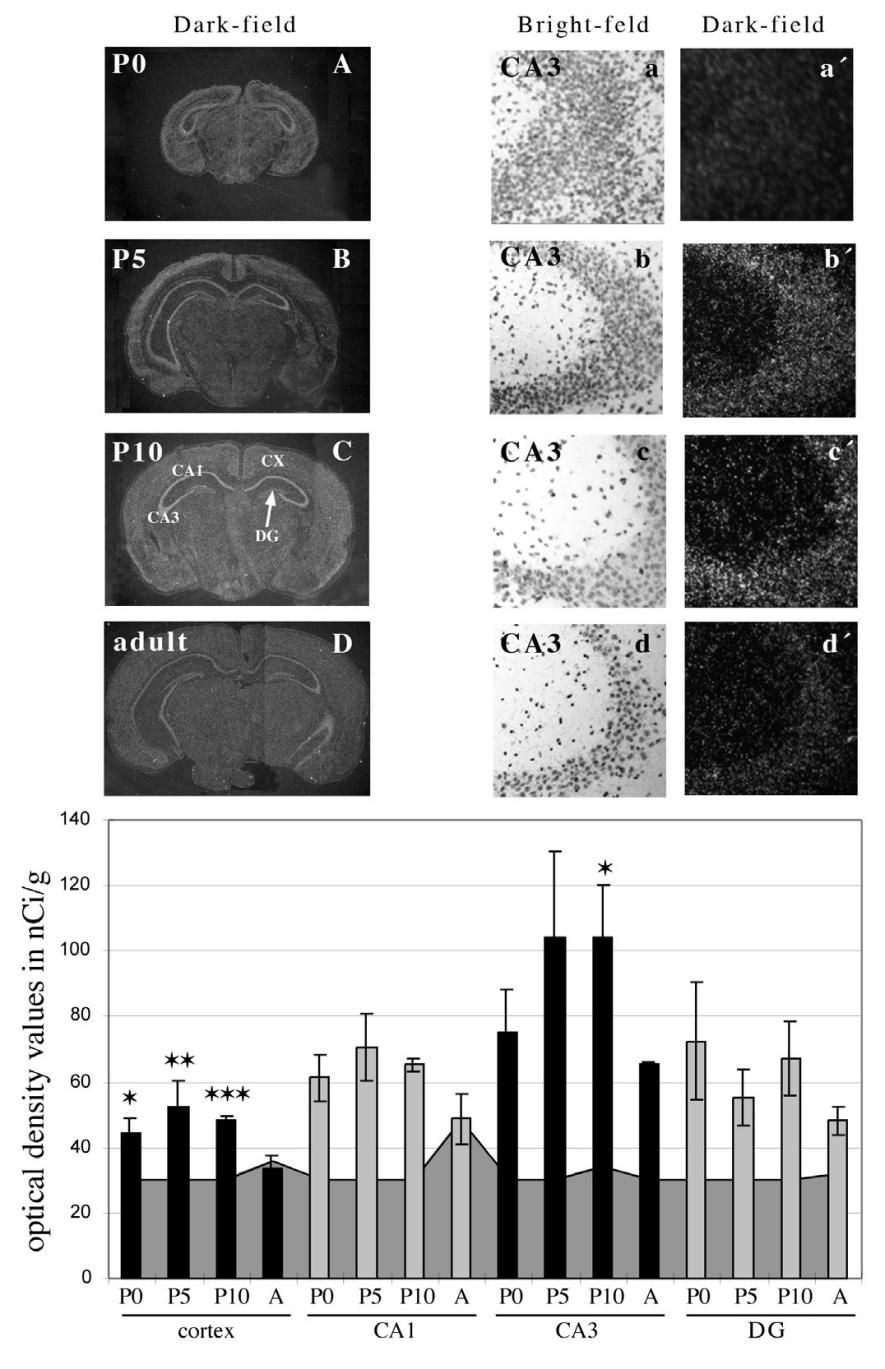
**Differential distribution of *Cln8 *mRNA in brains of P0, P5, P10 and adult mouse. **Dark-field emulsion autoradiographs from frontal sections, shown on the left, were analyzed by *in situ *hybridization analysis (A-D). Higher magnification in the hippocampal area CA3 is shown on the right. Bright-field views of the cells (a-d) and dark-field emulsion autoradiographs (a'-d') are shown. Optical density values of hybridization signals of *Cln8 *antisense probe are shown as bars in the diagram below (A = adult). Optical density values of hybridization signals of *Cln8 *sense probe are shown as a gray area behind the bars. In each brain region the optical density values at P0, P5 and P10 were compared to the optical density value of adult. Error bars represent standard error of the mean. Symbols *, ** and *** represent p < 0.05, 0.01 and 0.001, respectively. CX = cortex, DG = dentate gyrus

In postnatal mice (P0, P5, P10 and adult) *Cln8 *expression was quantitated in the cortex, cerebellum and different hippocampal regions. Expression at P0 was low but specific in hippocampal regions CA1, CA3 and the granular cell layer of the dentate gyrus and in the cortex (Fig. 3). At P5 and P10, the *Cln8 *expression increased in the hippocampal CA1 region (to 115% and 106% of P0) and especially in the CA3 region (to 139% of P0) (Fig. 3). In adult brain the expression levels of *Cln8 *were lower than at P0, P5 and P10 in every region analyzed (Fig. 3). When compared with adults, a statistically significant difference in *Cln8 *expression was detected in the cortex at P0, P5 and P10 and in the CA3 region at P10 (Fig. 3). Expression of *Cln8 *in adult cerebellum was 156% of expression in the cortex (data not shown). In all maturation stages, the expression of *Cln8 *was highest in hippocampal region CA3 (Fig. 3).

### *Cln8 *expression is strongly up-regulated in the hippocampal electrical kindling model of epilepsy

**Figure 4:**
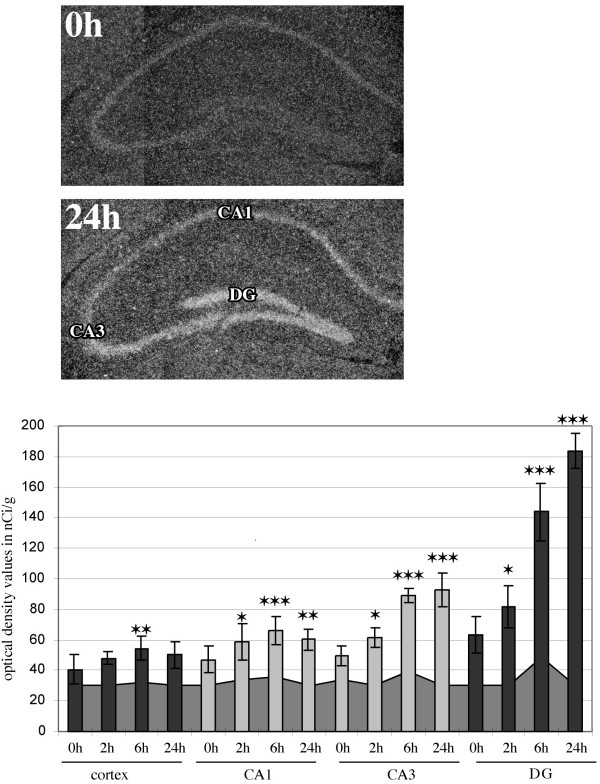
**Distribution of *Cln8 *mRNA in brains of hippocampal kindling induced epileptic mice. **Frontal mouse brain sections were analyzed 2 h, 6 h and 24 h after kindling induced epileptic seizures by *in situ *hybridization. Mice with electrodes implanted but without electrical stimulations were used as controls (0 h). Dark-field emulsion autoradiographs of hippocampal *Cln8 *expression in kindling-induced mice 0 h and 24 h after kindling are shown. Optical density values of hybridization signals of *Cln8 *antisense probe are shown as bars in the diagram. Optical density values of hybridization signals of *Cln8 *sense probe are shown as a gray area behind the bars. In each brain region the optical density values 2 h, 6 h and 24 h after kidling-induced seizures were compared to controls (0 h). Error bars represent standard error of the mean. Symbols *, ** and *** represent p < 0.05, 0.01 and 0.001, respectively. DG = dentate gyrus

Epileptic seizures are a characteristic feature in human patients with *CLN8 *mutations. We therefore analyzed, by mRNA *in situ *hybridization analysis, possible changes in *Cln8 *expression 2 h, 6 h and 24 h after kindling-induced epileptic seizures. Animals were subjected to 40 rapid kindling stimulations which lead to increased excitability in mice at 4 weeks after the stimulation [25]. Each rapid kindling stimulation induced relatively short focal (grade 0–2) or long-lasting generalized (grade 4–5) seizures. The mean duration of afterdischarges was 20 ± 3 s for focal seizures and 76 ± 8 s for generalized seizures. All animals experienced multiple (11 ± 2) grade 4–5 generalized tonic-clonic seizures. The expression of *Cln8 *in the control, electrode implanted but non-stimulated mice (0 h; Fig. 4) was essentially identical to that in wild-type adult mouse brain (Fig. 3). After kindling stimulations *Cln8 *was strongly up-regulated in the hippocampus, especially in the CA3 region and most prominently in the granular cell layer of the detate gyrus (Fig. 4). After 2 h, 6 h and 24 h expression of *Cln8 *in CA3 was 92%, 114% and 134% of that of controls, and in the granular cell layer of the dentate gyrus 128%, 225% and 286% of controls, respectively (Fig. 4). When compared to controls, a statistically significant increase in *Cln8 *expression was detected in the cortex after 6 h, and in the CA1 and CA3 regions, and the granular cell layer of the dentate gyrus after 2 h, 6 h and 24 h (Fig. 4). All changes in *Cln8 *mRNA expression after rapid kindling stimulations were bilateral with no differences between the sides.

## Discussion

We analyzed the expression of the *Cln8 *gene during mouse development and in the hippocampal kindling model of epilepsy to gain understanding of the role of CLN8 in the disease mechanisms underlying both human and mouse NCLs. In northern blot analysis a *Cln8 *transcript of approximately 3 kb was detected in all tissues studied. Two additional transcripts, approximately 2 kb and 7 kb, were detected in several tissues. Thus, *Cln8 *expression resembles that of human *CLN8*, which is ubiquitously expressed with transcripts of 1.4 kb, 3.4 kb and 7.5 kb [4]. The open reading frame of mouse *Cln8 *is 82% identical with *CLN8 *and at the amino acid level the proteins are 85% identical [4]. In both human and mouse genes the RT-PCR analysis of the *CLN8 *open reading frame resulted in a single fragment, suggesting that the different size transcripts seen in northern analysis are due to alternative 3' untranslated regions as previously suggested for the human *CLN8 *gene [4]. It is, however, also possible that the *CLN8*/*Cln8 *genes have several transcription initiation sites. Real-time quantitative RT-PCR analysis further suggested that the expression levels of *Cln8 *are relatively low in both adult and embryonic tissues, and showed that expression levels in various tissues do not differ dramatically. The remarkably low expression of *Cln8 *in testis could reflect a high level of the control gene analyzed in this tissue. Despite ubiquitous expression of the underlying gene and accumulation of storage material in all tissues, the most severe damage in EPMR patients and *mnd *mice is in neuronal cells. This is also characteristic of other NCLs, suggesting that NCL proteins may have a specific function in neuronal cells distinct from the role in non-neuronal cells, or that certain neuronal populations may be more sensitive to the disturbed function of NCL proteins.

*In situ *hybridization analysis of developing mice indicates that *Cln8 *mRNA is expressed throughout development as well as in the mature brain. The expression of *Cln8 *in embryonic stages E13 and E17 was specific but low in many tissues including brain, but remarkably high in the developing gastrointestinal tract. This may indicate a role for *Cln8 *in the innervation of the developing gut. In addition, in the E17 embryo *Cln8 *expression was high in DRG neurons. These neurons determine the connections between interneurons and motoneurons of the spinal cord. Assuming a role for Cln8 in this process, its disturbed function might result in defective connections in DRG with consecutive dysfunction of motor neurons resulting in progressive paralysis in the *mnd *mouse model [5,17]. The expression of *Cln8 *in postnatal mouse brain was highest at developmental stages P5 and P10, especially in hippocampal region CA3. The brain expression was clearly lower in adult mice than at P0, P5 and P10, suggesting a role for Cln8 in brain maturation. This early postnatal period is characterized by a rapid increase in the number of synapses, as well as maturation of oligodendrocytes, the myelin-forming cells in the CNS [26]. Two other NCL proteins, Ppt1 and Cln3, have been localized to synaptic areas of neurons [27,28] suggesting the importance of these NCL proteins for synaptic functions. However, detailed study of the function of Cln8 in the CNS and different brain cells is currently hampered by the lack of a specific antibody detecting endogenous Cln8. Very low levels of *Cln8 *mRNA were also detected in developing and adult motoneurons and retina (data not shown). However, the *Cln8 *transcript levels in these samples were virtually indistinguishable from the respective sense controls.

The expression of several other NCL genes in mouse and/or rat has been characterized. In addition to *Cln8, Cln1 *and *Cln5 *also show developmental regulation, whereas the expression of two other NCL genes, *cathepsin D *underlying a naturally occuring sheep NCL model and *Cln2 *underlying a human late infantile-onset NCL form, is relatively constant in the rat brain during development [29-32]. Mouse *Cln3 *is expressed in adult mice throughout the brain, and in the hippocampus the most prominent expression is detected in the granular cell layer of the dentate gyrus and pyramidal cells [28]. In addition, human *CLN1 *and *CLN5 *genes are expressed at the beginning of neurogenesis in embryonic human brains [33]. This expression increases as brain development proceeds. Expression of NCL genes, including *Cln8*, in the developing and mature brain indicates that NCL proteins may have roles not only in supporting the survival of neurons but also in the maturation and differentiation of different neuronal populations during development. Defects in maturation processes could potentially lead to neuronal degeneration and loss of neurons.

In the kindling model of experimental epilepsy a rapid up-regulation of *Cln8 *expression was detected in the brain, especially in hippocampal regions CA3 and the granular cell layer of the dentate gyrus. The expression of *Cln8 *increased at every time point measured, and was highest 24 h after kindling. Whether single or focal seizures alone are sufficient to induce *Cln8 *expression requires further investigation. While only *Cln8 *expression has been investigated in the kindling model, expression of *cathepsin D *and the *Cln1 *encoded palmitoyl protein thioesterase 1 (Ppt1) protein has been studied using kainic acid-induced seizures in rats as a model. Both show increased expression in this model, *cathepsin D *in the hippocampus, limbic cortex and temporo-parieto-occipital neocortex [34] and the Ppt1 protein most prominently in pyramidal cells of hippocampal regions CA1 and CA3 [35]. Although different forms of NCLs are genetically heterogenous, they share a common hippocampal pathology, which is distinct from lesions caused by anoxic-ischemic events and is instead suggested to be a consequence of primary metabolic defects [36]. Increased plasma glutamate levels, decreased glutamate uptake, descreased glutamate transporters as well as changes in ionotropic glutamate receptors has been described in *mnd *mice [37,38]. Also, prominent loss of GABAergic interneurons in *mnd *as well as in the *Cln1 *and *Cln3 *knock-out mice has been reported [39-41]. Alterations in both glutamatergic and GABAergic neurotransmission may both contribute to chronic excitotoxicity, hypothesized to underlie the cellular dysfunction and brain pathology in NCL disorders [42].

## Conclusion

The *Cln8 *gene is widely expressed in embryonic and adult mouse tissues. In line with this, and comparable with other NCL disorders, accumulation of storage deposits in CLN8-associated disorders occurs in all tissues. However, as the most striking accumulation and cellular dysfunction is limited to neurons, it is likely that CLN8/Cln8 is involved in a neuron-specific biochemical pathway or that neuronal cells are more sensitive to accumulating material or lack compensatory pathways.

The *Cln8 *gene is expressed throughout brain development and also in mature brain, suggesting a role for Cln8 in maturation and differentiation of neurons and in supporting the survival of neurons. The expression of *Cln8 *showed regional differences, suggesting a specific function for Cln8 in specific neuronal populations. The relevance of *Cln8 *up-regulation in hippocampal neurons of kindled mice needs to be studied further.

## Methods

### Northern blot analysis

FirstChoice™ Northern Blot Mouse Blot I (Ambion, Austin, TX, USA) was used to analyze the distribution of *Cln8 *expression in various tissues. An 872 base pair (bp) deoxyribonucleic acid (DNA) fragment including a *Cln8 *open reading frame of 867 bp, digested from the SvPoly-*Cln8 *expression vector with restriction enzyme *Bam*HI, was used as a probe. The hybridization was performed according to the manufacturer's instructions with Ultra sensitive Hybridization buffer (Ambion), and salmon sperm DNA and Cot-1 DNA for blocking.

### Real-time quantitative RT-PCR analysis of *Cln8*

Real-time quantitative RT-PCR analysis of *Cln8 *was performed in an ABI PRISM^® ^7000 SDS thermal cycler (Applied Biosystems, Foster City, CA, USA) using 5 μl of each tissue from Mouse Multiple Tissue cDNA (MTC™) Panel II (BD Biosciences) as template. Each 25 μl reaction contained 300 nM of reverse primer 5'-CCACTGGTTGGCCTTCCA-3' and 900 nM of forward primer 5'-GCCCTTCACCTGCATTTCC-3' as well as 200 nM of *Cln8 *specific probe 6FAM-TGACCACCCAGCCTTCAGGAGCAT-TAMRA in TaqMan^® ^Universal PCR Master Mix (Applied Biosystems). The primers were optimized for analysis and the PCR fragment of 76 bp covered the region from 510 to 585 bp of the *Cln8 *open reading frame (GenBank sequence accession no AF125307). The *Cln8 *specific probe included a reporter dye 6-carboxyfluorescein (FAM) and a quencher dye 6-carboxy-tetramethyl-rhodamine (TAMRA). Endogenous control reactions were performed using Assays-on-Demand Mm00446973_m1 TATA-box binding protein amplification according to the manufacturer's instructions (Applied Biosystems). Each reaction was performed in triplicate. Standard complementary DNA (cDNA) solutions, generated from C57/BL mice cortex mRNA, and standard curve method were used (User Bulletin #2: ABI PRISM(R) 7700 Sequence Detection System. P/N 4303859B (2001) Applied Biosystems. 36 s.). Statistical analyses were performed using Microsoft Excel.

The DNA fragment covering the whole open reading frame of *Cln8 *was PCR amplified using primers 3'-GTGATTTCTCCGGTGCTAGG-5' and 3'-GCAACCACCATTTCTCAGGT-5'.

### *In situ *hybridization

Paraffin and cryosections of NMRI or C57/BL mice were prepared and analyzed by mRNA *in situ *hybridization as previously described [43-45] with slight modifications. The isolated tissues from postnatal wild-type and kindling mice were fresh-frozen and sectioned. Embryos were either fixed in 4% paraformaldehyde and embedded in paraffin or fresh-frozen and sectioned. For all serial sections of 8–14 μm, a single ^35^S-labeled 370 bp *Cln8 *specific complementary RNA (cRNA) probe (from bp 29 to 398; GeneBank AF125307) was used. For the generation of the antisense and control sense cRNAs, the vector (pBluescript vector; Stratagene, La Jolla, CA, USA) containing the PCR-amplified *Cln8 *fragment was linearized and used as a template for RNA polymerases T3 and T7, respectively, using α-^35^S-labeled UTP (Amersham Biosciences, Uppsala, Sweden).

The paraffin sections were treated as cryosections, after first being deparaffinized in xylene and rehydrated in a descending ethanol series. All the slides were fixed in 4% paraformaldehyde, rinsed twice in phosphate-buffered saline and treated with proteinase K (20 μg/ml for paraffin sections, 1 μg/ml for cryosections). Next, the slides were postfixed in 4% paraformaldehyde, and the cryosections rinsed in 50% deionized formamide in 2 × standard sodium citrate (SSC). After being washed in distilled water, all slides were acetylated for 10 minutes with 0.25% acetic anhydride in 0.1 M triethanolamine. The paraffin sections were then dehydrated in an ascending ethanol series, while the cryosections were rinsed again with 50% formamide in 2 × SSC. Prehybridization for 1 h was subsequently performed at +52°C with hybridization buffer containing 60% formamide, 10% dextran sulphate, 1 × Denhardt's solution, 0.5 mg/ml yeast transfer RNA (tRNA), and 100 mM dithiothreitol. After prehybridization, cRNA probes were added to the hybridization buffer at a concentration of 35 000 cpm/μl, and hybridization was carried out overnight at +52°C in a chamber humidified with 60% formamide and 5 × SSC.

After hybridization the sections were first washed in 5 × SSC, 10 mM dithiothreitol at +50°C for 30 minutes, followed by 30 minutes in 50% formamide, 2 × SSC, 30 mM dithiothreitol at +65°C, and 3 × 10 minutes in NTE-buffer (0.5 M NaCl, 10 mM Tris-HCl, pH 8.0, 5 mM EDTA) at +37°C. Next, the slides were treated with RNase A (20 μg/ml) in NTE-buffer at +37°C for 30 min and washed for 15 min in NTE-buffer at +37°C. Washing with 50% formamide, 2 × SSC, 30 mM dithiothreitol was then repeated, after which the sections were washed in 2 × SSC and 0.1 × SSC, each for 15 min at +37°C. The slides were dehydrated at room temperature with ethanol containing 0.3 M ammonium acetate, air-dried, and exposed to BioMax MR Film (Kodak) together with ^14^C radioactive standards (Amersham Biosciences) for 5 days. Finally, the sections were dipped in NTB-2 emulsion (Kodak), developed after 3 to 4 weeks using D-19 developer (Kodak), fixed with Kodak Unifix, counterstained with hematoxylin (Shandon Inc./Thermoshandon, Pittsburgh, PA, USA), and mounted with Permount (Fisher Scientific International Inc., Hampton, NH, USA). Mice were bred in the Viikki Laboratory Animal Center, University of Helsinki. The experiments were conducted according to the "European Convention for the Protection of Vertebrate Animals used for Experimental and other Scientific purposes" with the approval of the institutional ethics committee.

### Rapid kindling procedure

Adult male C57/BL mice weighing 22–25 g (n = 16) were anaesthetized with sodium pentobarbital (60 mg/kg i.p.), and bipolar stainless steel electrodes (Plastics One) were implanted in the left ventral hippocampus using a Kopf stereotaxic frame as previously described [46]. One week later, the mice were electrically stimulated with forty threshold stimulations with 5 min intervals (10 Hz frequency, 1 ms square-wave pulses for 10 s). The electroencephalogram was continuously recorded during the whole rapid kindling procedure using the MacLab/4e system (ADInstruments). To determine the threshold, current intensity was increased stepwise (10 μA increments, starting from 10 μA) every 5 min until focal epileptiform activity (afterdischarge) of at least 5 s duration was elicited. Rapid kindling stimulations were then given on the same day. Behavioral convulsions were scored according to a modification [25] of the scale of Racine [47]: grade 0, arrest, normal behavior; grade 1, facial twitches; grade 2, chewing and nodding; grade 3, forelimb clonus; grade 4, rearing, body jerks, tail upholding; and grade 5, imbalance, hind-limb clonus and vocalization. At 2, 6 and 24 h after the last stimulus-evoked seizure, animals were decapitated (four animals in each group) and brains were processed for *in situ *hybridization. Four non-stimulated, electrode-implanted mice served as controls and were killed together with the experimental animals from different groups. The correct localization of the electrode was controlled for each animal in the coronal sections of the brain used for *in situ *hybridization. All experimental procedures were approved by the Research Ethics Committee at the Medical Faculty of the University of Lund.

### Quantitative and statistical analysis

Quantification of radioactive *in situ *hybridization films was done by digitizing the film images with a computer-based MCID image analysis system (Imaging Research). Gray levels from ^14^C radioactive standards were used in a third-degree polynomial calibration to obtain equivalent values of tissue radioactivity (nCi/g) for different brain regions. Measurements for each structure were carried out in several sections per animal. Since there were no significant differences between the two sides, values were pooled to obtain the mean value for each animal and brain region. Data from four control and four experimental animals for every probe and time point were used for analysis. Statistical analysis was performed using Student's t-tests. In the figures, the densities of hybridization signals of sense and antisense probes are shown separately. Sense values were not subtracted from antisense values because of different probes used. Error bars represent standard error of the mean; symbols *, ** and *** represent p < 0.05, 0.01 and 0.001, respectively.

## Authors' contributions

LL carried out real-time quantitative PCR and northern blot analyses. In addition, LL was involved in the analysis of mRNA *in situ *results and design of the study, drafted the manuscript and made the figures. AA carried out the mRNA *in situ *analysis and was involved in drafting the manuscript. OK prepared several mouse tissue samples for the mRNA *in situ *analysis, was involved in the analysis of the mRNA *in situ *results, and revised the manuscript. MK established the real-time quantitative RT-PCR methodology and helped with the analysis. ZK provided all kindling samples and contributed significantly to figures 3 and 4. mRNA *in situ *analyses were conducted under the supervision of MS, who also revised the manuscript and was involved in the design of the study. AEL was involved in the design of the study, analysis of the results and revision of the manuscript.
